# Efficacy of a Rapid Response Team on Reducing the Incidence and Mortality of Unexpected Cardiac Arrests

**DOI:** 10.5812/traumamon.4170

**Published:** 2012-07-31

**Authors:** Majid Sabahi, Seyed Ahmad Fanaei, Seyed Ali Ziaee, Farokh Sadat Falsafi

**Affiliations:** 1 Faculty of University of Sunny Brook, CA ATLS Instructor by American College of Surgeons, Toronto, Canada; 2 Department of Emergency Medicine, Atieh Hospital, Tehran, IR Iran; 3 Trauma Research Center, Baqiyatallah University of Medical Sciences, Tehran, IR Iran; 4 ATLS Instructor by American College of Surgeons, Emergency Department of Saudi German Hospital, Dubai, UAE; 5 Coordinator of Iran-ATLS Association, Atieh Hospital, Tehran, IR Iran

**Keywords:** Hospital, Hospital Rapid Response Team

## Abstract

**Background:**

Rapid Response Teams (RRTs) assess patients during early phases of deterioration to reduce patient morbidity and mortality.

**Objectives:**

This study aimed to evaluate the ability of earlier medical intervention by a RRT prompted by clinical instability in patients to reduce the incidence of and mortality from unexpected cardiac arrest at our hospital.

**Patients and Methods:**

A nonrandomized, population-based study before 2008 and after 2010 introduction of the Rapid Response Teams in a 300 bed private hospital. All patients were admitted to the hospital in 2008 (n = 25348) and 2010 (n = 28024). RRT (One doctor, one senior intensive care nurse and one staff nurse) attended to clinically unstable patients immediately with resuscitation drugs, fluid, and equipment. Response was activated by the bedside nurse or doctor according to predefined criteria. Main outcome measures were incidence and outcome of unexpected cardiac arrest.

**Results:**

The incidence of unexpected cardiac arrest was 17 per 1000 hospital admissions (431 cases) in 2008 (before RRT intervention) and 12.45 per 1000 admissions (349 cases) in 2010 (after intervention), with mortality being 73.23% (274 patients) and 66.15% (231 patients) respectively. After adjustment for case mix the intervention was associated with a 19% reduction in the incidence of unexpected cardiac arrest (odds ratio 0.81, 95% confidence interval 0.65-0.98).

**Conclusions:**

The RRT was able to detect preventable adverse events and reduce the mortality and incidence of unexpected cardiac arrests.

## 1. Background

Adverse events in hospitals associated with medical management are estimated to occur in 4.1% to 17.2 % of hospital admissions. Further evaluation and analyses of such events found that up to 70% of them were preventable (1, 2). One of the most dangerous and clinically considerable adverse events is unexpected cardiac arrest. Despite the availability of traditional cardiac arrest teams and advances in cardiopulmonary resuscitation, the risk of death from such an event has remained largely static (3, 4). Unexpected cardiac arrests in hospital are usually preceded by signs of clinical instability (5, 6). In a pilot study we noted that 112 (76%) patients with unexpected cardiac arrest or admission to intensive care had deterioration in the airway, circulation, or respiratory system for at least one hour (median 6.5 hours) before their index event (7). Despite this the hospital mortality for these patients was 62%.

Some studies around the world have demonstrated that patients admitted to hospitals suffer adverse events at a rate of between 2.9% and 17% of cases (8,9). Such events may not be directly related to the patient’s original diagnosis or underlying medical condition. Of greater concern, these events may result in prolonged length of hospital stay, permanent disability, and even death in up to 10% of cases. Other studies have shown that these events are frequently preceded by the signs of physiological instability that manifest as derangements in commonly measured vital signs (7, 10-12). Such derangements form the basis for Rapid Response Team (RRT) activation criteria used in many hospitals.

## 2. Objectives

In this study, we tested this hypothesis by conducting a prospective trial comparing these outcome measures before and after introducing a RRT.

## 3. Patients and Methods

We carried out a nonrandomised investigation in which the incidence of and mortality from cardiac arrest were recorded in inpatients at the Atieh Hospital over two 12-month periods: before 2008 and after 2010 (after the implementation of the intervention). Ethical approval for the study was granted from the Atieh Hospital ethics committee. Atieh Hospital is a 300 bed, general private hospital. Each year the emergency department treats about 120,000 patients, the hospital treats over 20,000 inpatients, and there are 500 to 600 admissions to the intensive care unit.

### 3.1. Implementation of RRP

In 2008 the hospital had a “traditional” system of response to clinically unstable patients. The nurse would observe and document the instability, a call would then be made to the most junior member of the medical team, who would attend the patient, review the problem, and institute treatment. If the patient’s condition continued to be unstable, the junior medical officer would seek advice from the next most senior member of the medical team concerned with the patient’s management (in our hospital, specialty registrar, internist). The treatment review cycle could then be repeated, often with referrals to other specialist services. Occasionally, these cycles were further repeated when the consultant reviewed the case and different teams of oncall doctors became involved. We gradually introduced the rapid response team into the hospital from 2010, using the same criteria as reported previously (10).

During 2010 we altered and completed the criteria for calling the team in response to feedback as an afferent limb from primary care nurses and senior medical officers (Table 1). The team was not called to the emergency department, operating theatres, intensive care or coronary care units. The criteria for RRT activation (Box 1) was displayed prominently in each ward. The RRT was activated by a pager call and by a public announcement internal communication call “C Code to Ward X”. The RRT initiated and completed a variety of therapeutic, investigational and procedural interventions (Table 2). Main outcome measures were incidence and outcome of unexpected cardiac arrest.

**Table 1:** Criteria for Calling Rapid Response Team

Airway	
Respiratory Distress, Wheezing, Congestion	
**Breathing**	
Respiratory Rate > 24 /min	Respiratory Rate < 8 /min
Saturation O2 < 90% on O2	Fio2 > 50%
**Circulation**	
Systolic Blood Pressure < 90 mm-Hg	HR < 40/min, HR > 130/min
Significant Bleeding	
**Neurologic**	
Changes in Consciousness	Seizure
**Other**	
Chest Pain	Uncontrolled pain
Restlessness	

**Table 2:** Interventions and procedures implemented by the Rapid Response Team

Interventions	
Nasopharyngeal/oropharyngeal suctioning and additional oxygen	Administration of IV a fluid bolus
Initiation of non-invasive positive pressure ventilation by mask	Nebulized medicine
Insertion of a Guedel airway	Administration of IV a sedative
Cardioversion and ongoing resuscitation	Acute transfusion of red cells
**Acute Investigations**	
Chest x-ray	Electrocardiogram
Arterial blood gases	Lab Test
**Invasive Procedures**	
IV a line insertion	Endotracheal intubation

^a^Abbreviation: IV: intravenous

## 4. Results

### 4.1. Cardiac Arrest

There were 25,348 admissions in the “before” period, compared with the 28,024 in the RRP intervention “after” period. The number of cardiac arrests in after patients decreased from 431 to 349 (RRR, 19%; P = 0.003). None of the patients suffering a cardiac arrest and receiving treatment had “do not resuscitate” orders explicitly written in the patient progress notes.

### 4.2. In-hospital Deaths

There were a total of 274 in-patient deaths in the “before” period compared with 231 deaths in the “after” period (RRR, 16%; P = 0.004, Table 3).

**Table 3:** Changes in Number of Cardiac Arrests and Mortality, Before and After Introducing the Rapid Response Team

	Before RRT a (2008)	After RRT a (2010)	Relative Risk Ratio (95% CI^a^)	Relative Risk Reduction, %
No. of cardiac arrests	431	349	0.81 (0.65-0.98)	19
In-patient deaths	274	231	0.84 (0.71-0.97)	16
Total admission	25348	28024		

^a^Abbreviations: CL: Confidence Interval, RRT: Rapid Response Team

## 5. Discussion

We found that the incidence of in hospital cardiac arrests decreased by 19% after the introduction of a RRT. In 1995, Lee et al. (13) published one of the first descriptions of the outcomes of using an RRT. In 1999, Goldhill et al. (14) reported that implementation of an RRT was associated with a 26% reduction in cardiac arrests before patients were transferred to the intensive care unit (ICU). Use of RRTs has resulted in a significant reduction in the number of codes called in units other than the ICU, as well as a decrease in the overall code rate in hospitals that use these teams (15-17). It is also consistent with previous observations that between 50% and 84% of in-hospital cardiac arrests are preceded by physiological instability (5-7, 17, 18).

Our study is the before-and-after study of RRT intervention that shows an impact on all causes of hospital mortality. This effect was only partly accounted for by the impact of the RRT on cardiac arrests. The RRT might therefore, confer other benefits, such as increasing awareness of the consequences of physiological instability. It is also possible that the educational program to introduce the RRT had an impact on the care of acutely ill patients. In contrast, a major multicenter, cluster-randomized, controlled trial called the Medical Early Response Intervention and Therapy (MERIT) (19) study failed to demonstrate a benefit. Moreover, the results of meta-analyses have questioned whether there are benefits and have suggested that further research is required (20, 21). It is important to consider our study’s limitations. Evidence supporting the effectiveness of rapid response systems comes from unblinded, nonrandomized, short-term studies at single centers, in which outcomes before and after the implementation of such systems were compared. These studies are subject to incorrect inferences about cause and effect or improved care with time (22). A recent before and after study of a nurse-led rapid-response team did not show a reduction in hospital codes or mortality (23). A meta-analysis by Chan et al. (23) concluded that “although RRTs [rapid-response teams] have broad appeal, robust evidence to support their effectiveness in reducing hospital mortality is lacking.” Similarly, a Cochrane meta-analysis (20) failed to confirm any benefit and suggested that “the lack of evidence on outreach requires further mult-center randomized, controlled trials to determine potential effectiveness.” Such trials are important for establishing the value of rapid-response systems in the prevention of serious adverse events in hospitals.

Our study demonstrating the effectiveness of RRTs on outcomes of in-hospital patients will be added to the current list. (Table 4) (24) Our RRT program has been successful in part because we have a dedicated, knowledgeable team who introduced, implemented, and evaluated the RRT to ensure that it is the best program possible. In addition, the implementation team became an interdisciplinary oversight team that continues to evaluate and improve the program on the basis of the evidence and recommendations from the RRT staff. Despite the controversy, we have a trained group of professionals, both in the RRT and in patient care units, whose aim is to render high-quality care for their patients.

**Table 4:** Summary of studies of Rapid Response Teams including comparative data (24)

	Study design	Findings a
Bristow *et al*. 2000 (25)	Case control cohort study comparison between one MET b hospital and two cardiac admissions in MET b hospital. No difference in arrest team hospitals in-hospital cardiac arrests or mortality	Fewer unanticipated ICU b /high dependency unit
Buist *et al*. 2002 (26)	Before (1996) and after (1999) study MET b introduced in 1997 and criteria simplified 1998	Reduction of cardiac arrest rate from 3.77 to activation 2.05/1,000 admissions. OR b for cardiac arrest after adjustment for case mix = 0.50 (95% CI 0.35 to 0.73)
Bellomo *et al.* 2003 (27)	Before (4 months 1999) and after (4 months 2000 to 2001) 1- year preparation and education period	RRR b cardiac arrests 65% (*P *< 0.001). Decreased bed and days cardiac arrest survivors (RRR b 80%, *P *< 0.001). Reduced hospital mortality (RRR b 26%, *P *= 0.004)
Bellomo *et al.* 2004 (28)	Time periods and design as above. Assessment of effect of MET b on serious adverse events following major surgery	Reduction in serious adverse events (RRR 57.8%, *P *< 0.001), emergency ICU b admissions (RRR b 44.4%, *P *= 0.001), postoperative deaths (RRR b 36.6%, *P *= 0.0178), and hospital length of stay (*P *= 0.0092)
Kenward *et al*. 2004 (29)	Before and after (October 2000 to September 2001) introduction of MET ^b^	Decreased deaths (2.0% to 1.97%) and cardiac arrests (2.6/1,000to2.4/1,000admissions). Not significant
DeVita *et al*. 2004 (30)	Retrospective analysis of MET b activations and cardiac arrests over 6.8 years	Increased MET b use (13.7 to 25.8/1,000 admissions) was associated with 17% reduction cardiac arrests(6.5 to 5.4/1,000 admissions, *P *= 0.016)
Priestly *et al*. 2004 (31)	Single-centre ward-based cluster randomized control trial of 16 wards.	Critical care outreach reduced in-hospital mortality (OR b 0.52, 95% CI b 0.32 to 0.85) compared with control wards.
MERIT 2005 (19)	Cluster randomized trial of 23 hospitals in which 12 introduced a MET b and 11 maintained only a cardiac arrest team. Four-month preparation period and 6-month intervention period	Increased overall call rates (3.1 *vs.* 8.7/1,000 admissions, *P *= 0.0001). No decrease incomposite end point of cardiac arrests, unplanned ICU b admissions and unexpected deaths
Jones *et al*. 2005 (32)	Long-term before (8 months 1999) and after (4 years) introduction of MET b	Decreased cardiac arrests (4.06 to 1.9/1,000 admissions; OR b 0.47, P < 0.0001). Inverse correlation between MET b rate and cardiac arrest rate (r2 0.84, P = 0.01)
Jones *et al*. 2007 (33)	Long-term before (September 1999 to August 2000) and after (November 2000 to December 2004) study. Effect on all-cause hospital mortality	Reduced deaths in surgical patient compared with ‘before’ period (P = 0.0174). Increased deaths in medical patients compared with ‘before’ period (P < 0.0001)
Jones *et al*. 2007 (34)	Time periods of design as per [29]. Study assessed long-term (4.1 years) survival of major surgery cohort	Patients admitted in the MET b period had a 4.1-year survival rate of 71.6% versus 65.8% for control period. Admission during MET b period was an independent predictor of decreased mortality (OR b 0.74, *P *= 0.005)
Buist *et al*. 2007 (35)	Assessment of MET b call rates and cardiac arrests between 2000 and 2005	Increased MET b use was associated with reduction in cardiac arrest of 24% per year, from 2.4 to 0.66/1,000admissions
Jones *et al*. 2008 (36)	Multi-centre before-and-after study.Assessment of cardiac arrests admitted from ward to ICU b before and after introduction of RRT b	Continuous data only available for one-quarter of 172 hospitals. Temporal trends suggest reduction in cardiac arrests in both MET b and non-MET b hospitals
Chan *et al*. 2008 (23)	18-month-before and 18-month-after study following introduction of RRT b	Decrease in mean hospital codes (11.2 to 7.5/1,000 admissions) but not significant after adjustment (0.76 (95% CI b, 0.57 to 1.0); *P *= 0.06). Lower rates of non-ICU b codes (AOR b 0.59 (95% CI b, 0.40 to 0.89) versus ICU b codes AOR b, 0.95 (95% CI b, 0.64 to 1.43); *P *= 0.03 for interaction). No decrease in hospital-wide mortality 3.22% versus 3.09% (AOR b, 0.95 (95% CI b, 0.81 to 1.11); *P *= 0.52)
Sabahi *et al.* 2011	12-month-before and 12 month-after study following introduction of RRT b	Decreased cardiac arrests (17 to 12.5/1,000 admissions; OR b 0.74, *P *< 0.0001). Decreased deaths in admitted patients compared with ‘before’ period(*P *< 0.0001)

^a^Comparative data refer to before and after, contemporaneous case control or cluster randomized controlled trial

^b^Abbreviations: AOR: Adjusted Odds Ratio, CI: Confidence Interval, ICU: Intensive Care Unit, MET: Medical Emergency Team, OR: Odds Ratio, RRR: Relative Risk Reduction, RRT: Rapid Response Team
